# A prospective real-world study of efgartigimod in the treatment of chronic inflammatory demyelinating polyradiculoneuropathy

**DOI:** 10.3389/fimmu.2026.1779544

**Published:** 2026-03-02

**Authors:** Xiangtao Nie, Gen Huang, Yongbo Ma, Geke Zhu, Wenjing Qi, Han Zhou, Wanying Wang, Lei Hao, Xiuming Guo

**Affiliations:** 1 Department of Neurology, The First Affiliated Hospital of Chongqing Medical University, Chongqing, China; 2 Department of Neurology, People’s Hospital of Chongqing Hechuan, Chongqing, China; 3 Department of Neurology, Bazhong Central Hospital, Bazhong, Sichuan, China

**Keywords:** adverse events, chronic inflammatory demyelinating polyradiculoneuropathy, efgartigimod, real-world study, treatment

## Abstract

**Objective:**

To evaluate the short-term clinical efficacy and safety of efgartigimod in the treatment of Chronic Inflammatory Demyelinating Polyradiculoneuropathy (CIDP) in a real-world setting.

**Methods:**

This prospective real-world study enrolled 12 CIDP patients receiving efgartigimod treatment at the Department of Neurology, The First Affiliated Hospital of Chongqing Medical University. Efficacy was comprehensively assessed using the Medical Research Council (MRC) scale for muscle strength, the inflammatory Rasch-built Overall Disability Scale (I-RODS) for disability, and the Inflammatory Neuropathy Cause and Treatment (INCAT) scale. Assessments were performed before the first dose and one week after each subsequent dose. Adverse events during treatment, concomitant use of other immunosuppressants, routine laboratory tests, electrocardiograms, and immunoglobulin levels before and after treatment were recorded.

**Results:**

Among the 12 patients, 50% received subcutaneous injection and 50% received intravenous infusion of efgartigimod. During the follow-up period, significant efficacy was observed overall, MRC scores increased from 45.92 ± 15.69 to 55.00 ± 10.14, I-RODS scores increased from 49.25 ± 24.21 to 75.42 ± 28.46, and INCAT scores decreased from 4.42 ± 3.20 to 1.42 ± 2.61. By the end of the follow-up at the fifth week after the first dose, 11 patients (91.7%) achieved clinical improvement. No significant difference in efficacy was found between the subcutaneous and intravenous administration routes. Laboratory tests showed a downward trend in IgG levels after treatment, with no significant decrease in albumin. The incidence of adverse events during treatment was low, with only one patient experiencing a localized rash.

**Conclusion:**

This study demonstrates the short-term efficacy and safety of efgartigimod in treating CIDP, suggesting its potential as a safe and effective alternative therapy. Further exploration is needed to determine its suitability for long-term maintenance treatment of CIDP.

## 1 Introduction

Chronic inflammatory demyelinating polyradiculoneuropathy (CIDP) is an immune-mediated peripheral neuropathy characterized by demyelination and axonal damage of peripheral nerves (1). Patients present with progressive limb weakness and sensory disturbances, with symptoms progressing for at least 8 weeks, often in a relapsing-remitting pattern (2). Without treatment, it can lead to disability significantly impacting quality of life (3). First-line treatments recommended by international guidelines include corticosteroids, intravenous immunoglobulin (IVIG), and plasma exchange (PE). However, these options have limitations, and some patients do not achieve complete remission even after prolonged first-line therapy (2). Therefore, new treatment options are urgently needed.

The pathophysiological mechanism of CIDP is not fully understood. Previous studies suggest involvement of both cellular and humoral immunity, with autoantibodies playing a significant role (1, 4–6). Basic research shows that CIDP disease models can be established via antibody- or antigen-mediated immune responses. Multiple clinical studies confirm the efficacy of antibody-depleting therapies like IVIG, PE, and immunoadsorption (IA) in treating CIDP (7–10), indicating the potential pathophysiological role of autoantibodies. Thus, CIDP can be defined as an antibody-mediated autoimmune disease. Reducing circulating levels of pathogenic IgG antibodies is a key therapeutic strategy.

The neonatal Fc receptor (FcRn) plays a crucial role in maintaining IgG homeostasis. Through FcRn-mediated recycling, IgG is protected from intracellular degradation and transported extracellularly, maintaining high serum concentrations and prolonging its half-life (11–14).

Efgartigimod is the first globally approved FcRn inhibitor. It competitively binds to FcRn, reducing IgG recycling and thereby clearing pathogenic IgG antibodies, providing targeted therapy for neuroimmune diseases. Compared to traditional treatments, it specifically targets IgG without affecting other antibodies or cytokines, showing promising potential in antibody-mediated neuroimmune disorders (15–17). In a phase III clinical trial (ADAPT) for generalized myasthenia gravis (gMG), efgartigimod demonstrated favorable clinical efficacy and safety (18). Furthermore, efgartigimod has shown preliminary positive results in CIDP patients. A multicenter phase II trial (ADHERE) in CIDP patients demonstrated its effectiveness, tolerability, safety, and ability to reduce relapse risk (19). Real-world clinical studies have also corroborated efficacy and safety consistent with trial results (20–24). The intravenous formulation of efgartigimod was approved in China in September 2023 for treating adult AChR-positive gMG patients, and the subcutaneous formulation was approved in November 2024 for treating adult AChR-positive gMG and CIDP patients. However, real-world studies on this novel drug for CIDP remain scarce, with limited clinical experience.

This single-center prospective real-world study conducted at The First Affiliated Hospital of Chongqing Medical University aims to evaluate the short-term clinical efficacy and safety of efgartigimod in the Chinese CIDP population, providing more real-world experience for clinicians.

## 2 Methods

### 2.1 Study design

This is a prospective real-world study conducted from January 2024 to November 2025 at The First Affiliated Hospital of Chongqing Medical University. CIDP patients from the inpatient and outpatient departments of Neurology were screened and enrolled. The study aimed to explore the short-term clinical efficacy and safety of efgartigimod in the Chinese CIDP population and provide clinical experience. All enrolled patients met the diagnostic criteria for CIDP according to the European Federation of Neurological Societies/Peripheral Nerve Society (EFNS/PNS) consensus guidelines (second revision) (2) and had a CIDP Disease Activity Status (CDAS) score ≥ 2. Exclusion criteria were: (1) other chronic immune system diseases, either active or stable but requiring corticosteroid treatment (e.g., rheumatoid arthritis, scleroderma, ulcerative colitis); (2) other severe comorbidities (e.g., active hepatitis, tuberculosis, HIV, syphilis, malignant tumors); (3) positive for anti-paranodal antibodies. The study was conducted in accordance with the ethical principles of the Declaration of Helsinki and approved by the Ethics Committee of The First Affiliated Hospital of Chongqing Medical University (Approval No.: 2025-735-01). Informed consent was obtained from all patients or their families.

### 2.2 Treatment

Based on clinical symptoms, patient preference, and neurologist’s experience, patients received efgartigimod via intravenous or subcutaneous administration once weekly for four consecutive doses. The intravenous dose was 10 mg/kg per infusion, and the subcutaneous dose was 1000 mg per injection, adjustable based on patient condition. Concomitant corticosteroids or other immunosuppressants were allowed. Patients requiring a change in treatment due to severe adverse events or significant disease worsening were withdrawn from the efficacy analysis but followed for prognosis.

### 2.3 Data collection

Baseline data collected at enrollment included age, sex, body mass index (BMI), CIDP subtype, mode of onset, time from onset to initiation of induction therapy, disease duration, cerebrospinal fluid results, nerve conduction study results, comorbidities, and prior medications. Disease severity was assessed using the Medical Research Council (MRC) scale, the inflammatory Rasch-built Overall Disability Scale (I-RODS), and the Inflammatory Neuropathy Cause and Treatment (INCAT) scale at baseline and one week after each dose to evaluate changes in daily activity and functional disability. Scores at each time point were compared to baseline to calculate changes. Immunoglobulin levels (IgG, IgA, IgM), complete blood count, liver and kidney function, lipid profile, stool and urine routine, and electrocardiogram were measured before and after efgartigimod treatment.

### 2.4 Efficacy assessment

Treatment response was determined based on EFNS/PNS guidelines and related clinical studies (2). “Improvement” was defined as: an increase in MRC score by ≥2 points from baseline and/or an increase in I-RODS score by ≥4 percentile points from baseline and/or a decrease in INCAT score by ≥1 point. Efficacy measures included the proportion of patients achieving “improvement” and changes from baseline in the three scores (INCAT, I-RODS, MRC), along with changes in IgG, IgA, and IgM levels.

### 2.5 Safety evaluation

Adverse events (AEs) and serious adverse events (SAEs) during and after efgartigimod treatment were recorded. Common AEs included headache, nausea, diarrhea, respiratory or urinary tract infections, rash, etc. Vital signs were monitored. Serum albumin and lipid levels before treatment and at the last follow-up were also recorded.

### 2.6 Statistical analysis

Statistical analysis was performed using IBM SPSS Statistics 25. Raw I-RODS scores were converted to percentile intervals using Rasch analysis before evaluation. MRC and INCAT scores were analyzed as raw data. Categorical variables are presented as frequencies and percentages. Normally distributed continuous variables are presented as mean ± standard deviation, and non-normally distributed continuous variables as median (interquartile range). Continuous variables were compared using independent samples t-test or Mann-Whitney U test, and categorical variables using chi-square test. A p-value < 0.05 was considered statistically significant.

## 3 Results

### 3.1 Patient demographics and clinical characteristics

The study enrolled 12 eligible CIDP patients, all with typical CIDP. These patients opted for efgartigimod due to clinical worsening. Six patients received subcutaneous injection and six received intravenous infusion. **Table 1** summarizes the main demographic and baseline characteristics. The cohort included 7 females and 5 males, with a mean age of 46.67 ± 18.27 years and a median disease duration of 12 (3.25- 36.00) months. Seven patients had acute onset (A-CIDP), and five had chronic onset. Lumbar puncture showed albuminocytologic dissociation in 9 patients. Nerve conduction studies indicated secondary axonal damage in 8 patients. Two patients had other autoimmune diseases: one with undifferentiated connective tissue disease and one with autoimmune hepatitis. As shown in **Table 2**, most patients had received prior immunotherapies for CIDP. Among the 7 A-CIDP patients, 4 had received IVIG followed by steroids, 1 had received PE followed by IVIG, 1 had received PE followed by rituximab, and 1 had received IVIG alone. Five patients were on immunosuppressants at enrollment: 3 on corticosteroids and 2 on tacrolimus.

**Table 1:** Patient demographic and clinical characteristics.

Characteristic	All patients(n=12)	Intravenous(n=6)	Subcutaneous(n=6)	*P* value
Age, years	46.67(18.27)	37.83(18.10)	55.50(14.82)	0.094
Gender				1.000
female	7(58%)	3(50%)	4(67%)	
male	5(42%)	3(50%)	2(33%)	
BMI, kg/m²	22.99(3.54)	22.95(3.80)	23.03(3.64)	0.970
Onset type				1.000
Acute	7(58%)	4(67%)	3(50%)	
Chronic	5(42%)	2(33%)	3(50%)	
Disease Duration, months	12(3.25-36.00)	26(10.00-78.00)	6.50(2.75-18.00)	0.053
Albuminocytologic dissociation	9(75%)	4(67%)	5(83%)	1.000
Axonal damage	8(67%)	5(83%)	3(50%)	0.545
Prior treatment				
IVIg	7(58%)	4(67%)	3(50.0%)	1.000
Corticosteroids	5(42%)	4(67%)	1(17%)	0.242
PE	4(33%)	3(50%)	1(17%)	0.545
Rituximab	2(17%)	1(17%)	1(17%)	1.000
INCAT score	4.42(3.20)	3.67(2.94)	5.17(3.55)	0.444
MRC score	45.92(15.69)	48.17(14.57)	43.67(17.82)	0.642
I-RODS score	49.25(24.21)	53.83(20.67)	44.67(28.49)	0.538

Data are mean (SD), n (%) or median (IQR).

BMI, Body Mass Index; IVIg, intravenous immunoglobulin; PE, Plasma exchange; MRC, medical research council; I-RODS, inflammatory Rasch-built overall disability scale; INCAT, Inflammatory Neuropathy Cause and Treatment.

**Table 2:** Treatment regimens of patients.

Patient No.	Onset type	Time from onset to initial treatment	Prior treatment	Efficacy of prior treatment	Concomitant medications
P1	Acute	21 days	IVIG, GCs	Effective	GCs
P2	Chronic	2 years	GCs, PE	Effective	GCs
P3	Acute	2 months	IVIG, GCs	Effective	HCQ
P4	Acute	6 months	IVIG, GCs	Effective	Tac
P5	Acute	2 months	IVIG, PE	Effective	–
P6	Chronic	1 year	PE, RTX	Effective	–
P7	Acute	1 months	IVIG	None	–
P8	Acute	10 days	IVIG, GCs	Effective	GCs
P9	Chronic	2 years	Tac	Effective	Tac
P10	Acute	13 days	PE, RTX	Effective	–
P11	Chronic	1 year	–	Effective	–
P12	Chronic	8 months	IVIG	Effective	MMF

IVIG, intravenous immunoglobulin; GCs, Corticosteroids; PE, plasma exchange;RTX, rituximab;Tac, Tacrolimus; HCQ, hydroxychloroquine; MMF, mycophenolate mofetil.

### 3.2 Treatment regimens

Efgartigimod regimens varied based on clinical practice. Patient 4 did not receive the fourth dose because symptoms had largely resolved after three treatments. Patient 12, with a long disease duration, showed poor response to efgartigimod. Mycophenolate mofetil was added after the second dose, but after three doses with no significant improvement, efgartigimod was discontinued. Treatment was switched to IVIG, followed by oral corticosteroids and mycophenolate mofetil for maintenance. Patient 7, with electrophysiological evidence of severe combined demyelinating and axonal damage, achieved only partial remission after four doses. Although meeting the “improvement” criteria, the INCAT score remained at 3, so the weekly treatment plan was extended to eight weeks.

### 3.3 Efficacy assessment

**Figure 1:**
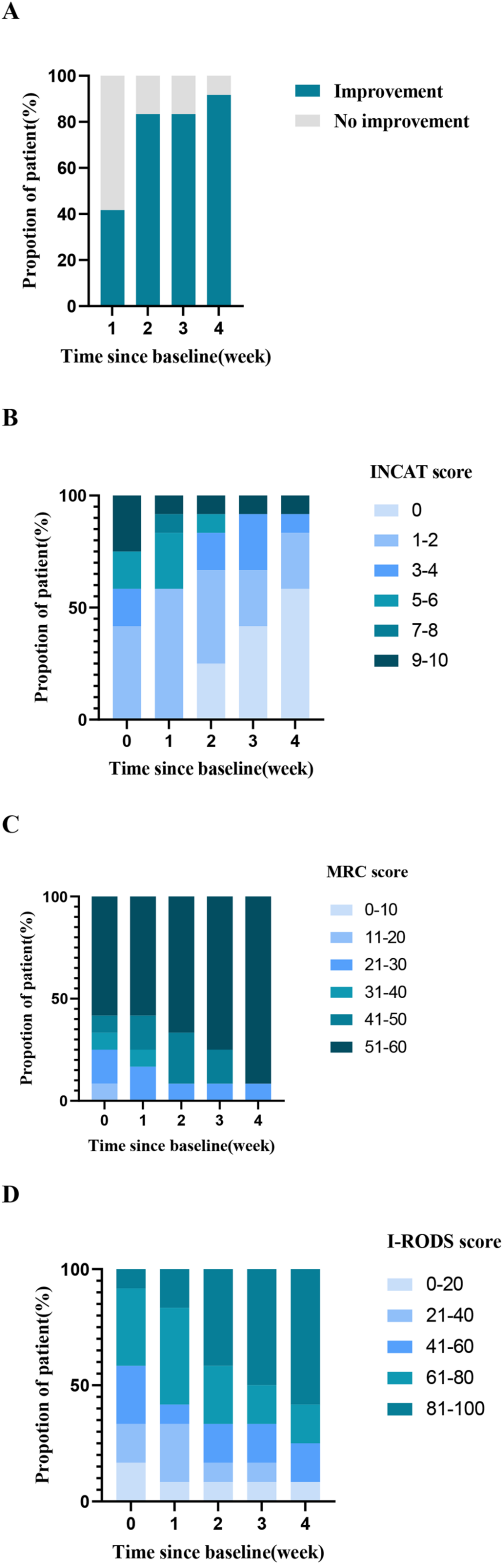
**(A)** Percentage of patients who achieved improvement changed over time following efgartigimod treatment. “Improvement” was defined as: an increase in MRC score by ≥2 points from baseline and/or an increase in I-RODS score by ≥4 percentile points from baseline and/or a decrease in INCAT score by ≥1 point. **(B)** The cumulative percentage histogram illustrates the proportion of patients with each INCAT score at baseline and at one-week follow-up after each efgartigimod treatment. The vertical axis represents the percentage of patients corresponding to INCAT scores ranging from 0 to 10. **(C)** The cumulative percentage histogram illustrates the proportion of patients with each MRC score at baseline and at one-week follow-up after each efgartigimod treatment. The vertical axis represents the percentage of patients corresponding to MRC scores ranging from 0 to 60. **(D)** The cumulative percentage histogram illustrates the proportion of patients with each I-RODS score at baseline and at one-week follow-up after each efgartigimod treatment. The vertical axis represents the percentage of patients corresponding to I-RODS scores ranging from 0 to 100. INCAT, Inflammatory Neuropathy Cause and Treatment; MRC, medical research council; I-RODS, inflammatory Rasch-built overall disability scale.

**Figure 2:**
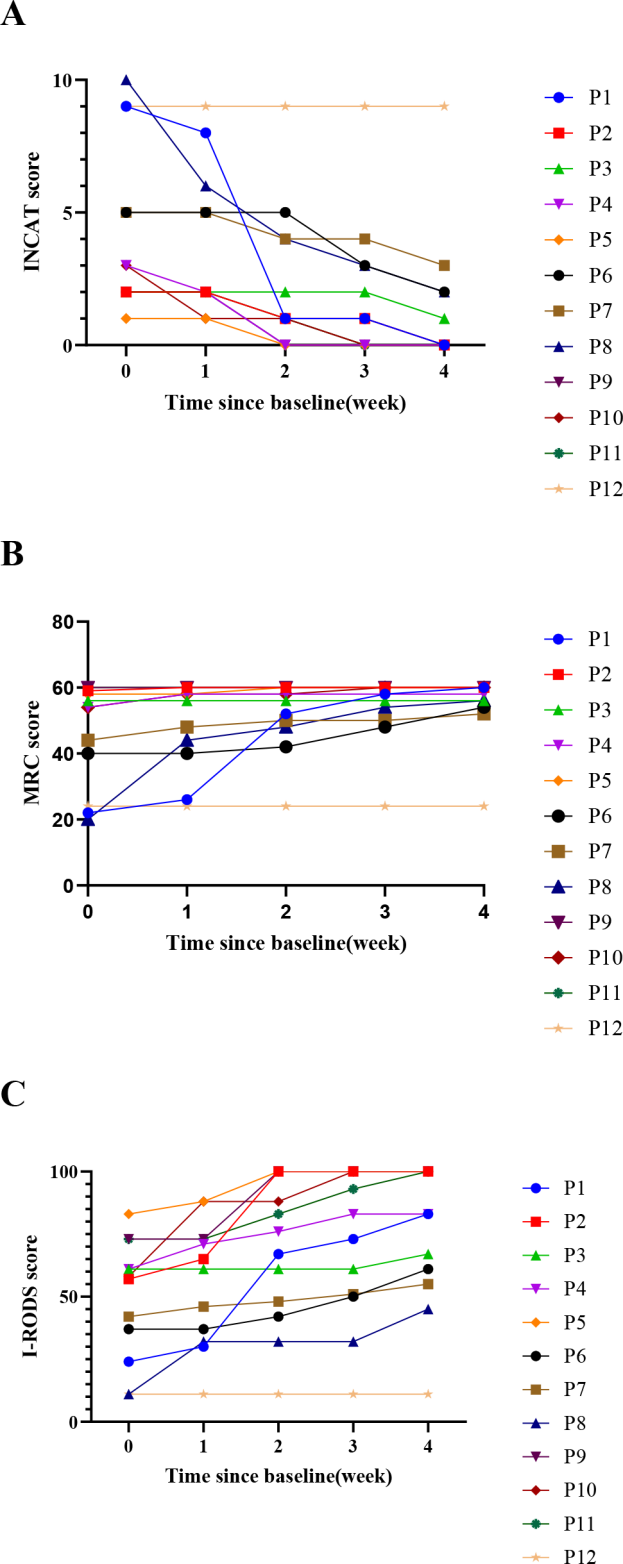
These figures illustrate patients’ daily activity and functional disability status, as quantified by the INCAT, I-RODS, and MRC scales, at baseline and at one week after each efgartigimod infusion. **(A)** INCAT Scores; **(B)** MRC scores; **(C)** I-RODS Scores. INCAT, Inflammatory Neuropathy Cause and Treatment; MRC, medical research council; I-RODS, inflammatory Rasch-built overall disability scale.

**Figure 3:**
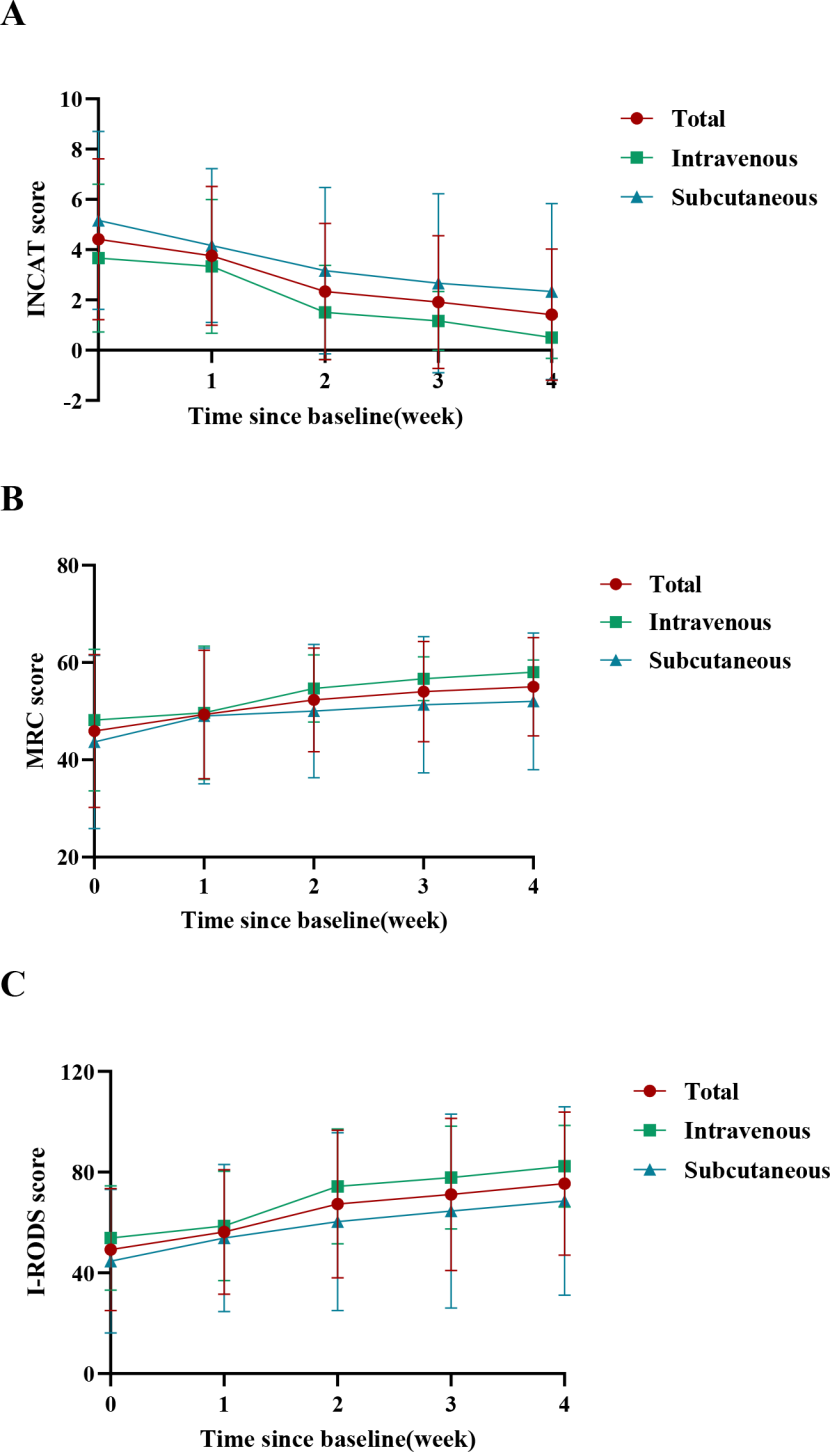
Longitudinal changes in clinical scores over time for the overall patient cohort, as well as separately for patients receiving intravenous versus subcutaneous administration. **(A)** INCAT Scores; **(B)** MRC scores; **(C)** I-RODS Scores. INCAT, Inflammatory Neuropathy Cause and Treatment; MRC, medical research council; I-RODS, inflammatory Rasch-built overall disability scale.

During follow-up, enrolled patients showed significant overall efficacy (**Figure 1**). One week after the first dose, 5 patients (42%) achieved clinical improvement (2 IV, 3 SC). One week after the second dose, 10 patients (83%) improved (5 IV, 5 SC). One week after the fourth dose, 11 patients (92%) improved (6 IV [100%], 5 SC [83%]). **Figure 2** presents the scores of the 12 patients at each follow-up time point. As shown in **Figure 3**, compared with baseline, MRC scores increased from 45.92 ± 15.69 to 55.00 ± 10.14 at the final follow-up, I-RODS scores increased from 49.25 ± 24.21 to 75.42 ± 28.46, and INCAT scores decreased from 4.42 ± 3.20 to 1.42 ± 2.61. Only one patient in the subcutaneous group did not meet the improvement criteria.

Furthermore, the 7 A-CIDP patients responded well to efgartigimod: 5 patients (71%) improved after the first dose, and all improved after the fourth dose. Among the 5 chronic-onset patients, none (0%) improved after the first dose, but 4 (80%) improved at the follow-up one week after the fourth dose.

Comparative analysis between intravenous and subcutaneous administration routes showed that MRC and I-RODS scores increased, and INCAT scores decreased over time in both groups(**Figure 3**), the scores showed no statistically significant difference between groups (p>0.05, **Supplementary Tables S1**–**3**).

### 3.4 Safety assessment

**Figure 4:**
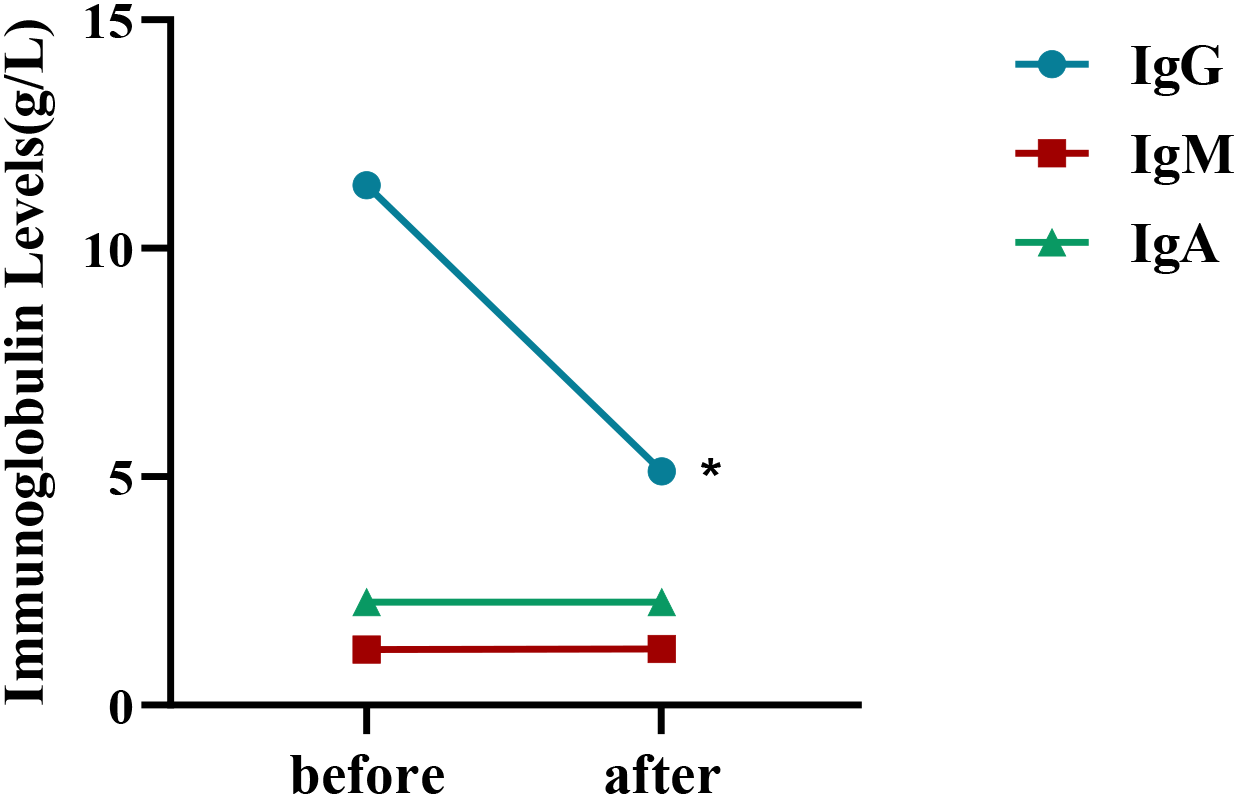
Changes in immunoglobulin levels before and after treatment. Serum levels of IgG, IgM, and IgA were assessed before and after treatment in nine patients. “before” denotes the time point immediately before the initial administration of efgartigimod, while “after” indicates the time point at the final follow-up. *p<0.05. Error bars show standard error.

Most patients had IgG antibody levels measured before and after treatment (**Supplementary Table S4**). Available data from 9 patients showed decreased IgG levels during treatment compared to prior measurements in 8 patients. One patient had low baseline IgG, and no concentration decrease was observed post-treatment; IgG fluctuation was around 5 g/L. Overall, there was a statistically significant difference in IgG levels before and after treatment (p<0.05, **Figure 4**). IgM, IgA, and albumin levels showed no significant decrease(p>0.05, **Figure 4**), and LDL-C levels showed no significant increase. Only one adverse event was observed: a rash on the anterior chest and neck during the third infusion, which resolved spontaneously after stopping the infusion and did not recur during the fourth dose. No other AEs such as headache, vomiting, diarrhea, or respiratory/urinary tract infections were observed. No patient required dose reduction or discontinuation due to AEs. No serious adverse events occurred.

### 3.5 Concomitant medications

Only three patients(Patients 1, 2, and 8) continued oral corticosteroid therapy during efgartigimod treatment, with gradual tapering during follow-up. Upon enrollment, Patient 1 was receiving prednisone at a dose of 25 mg/day, which was being tapered at a rate of 5 mg every two weeks. This tapering regimen was continued after the initiation of efgartigimod. Following the complete discontinuation of glucocorticoids, maintenance therapy was switched to mycophenolate mofetil. At the time of enrollment, Patients 2 and 8 were on maintenance therapy with prednisone 20 mg/day. After adding efgartigimod, their prednisone dose was reduced at a speed of 5 mg every two weeks. Patients 4 and 9 received concomitant oral tacrolimus. Patient 12 received mycophenolate mofetil after two subcutaneous efgartigimod doses. Due to inadequate response, the fourth efgartigimod dose was omitted, but mycophenolate mofetil was continued.

## 4 Discussion

Since its approval, efgartigimod has been primarily used for immunotherapy in MG patients. With increasing patient benefit, CIDP has also been included in its indications. Currently, there are few reports on efgartigimod for CIDP in Chinese patients. A small-sample prospective observational case series from Huashan Hospital, Fudan University, China, showed good short-term efficacy and safety of efgartigimod in CIDP (22). Additionally, satisfactory results have been reported in real-world studies for other neurological autoimmune diseases like MG and immune-mediated necrotizing myopathy (IMNM) (20, 24, 25). This study enrolled 12 CIDP patients, evaluating the efficacy and safety of efgartigimod in a real-world clinical setting, providing valuable clinical experience.

Autoimmune antibodies are widely considered key in CIDP pathophysiology (4, 26, 27). FcRn inhibitors reduce serum IgG recycling and are used to treat various IgG-induced autoimmune diseases (12). Efgartigimod binds with high affinity to FcRn, enhancing IgG degradation (15, 28). The ADHERE study showed that 66% of patients had evidence of clinical improvement (ECI) after efgartigimod treatment in the open-label A phase. In the randomized double-blind B phase, efgartigimod significantly reduced the risk of CIDP relapse compared to placebo, with clinical benefits observed across all efficacy scales and patient subgroups regardless of prior treatment (19).

The ADHERE study demonstrated rapid onset of action for efgartigimod, with 40% (128/322) of patients showing ECI at week 4, the earliest possible time point. Our study included a broader patient population. In these patients, efgartigimod showed significant short-term efficacy, with the vast majority (91.7%) achieving clinically meaningful improvement after four weeks of treatment. MRC and I-RODS scores improved significantly, indicating marked effectiveness during the induction phase. Notably, 5 patients (41.7%) achieved clinical improvement as early as one week after the first dose, highlighting the importance of efgartigimod for early symptom relief. Most patients in our study had received prior immunotherapies. Five patients experienced relapse despite regular corticosteroid or tacrolimus use, but showed significant improvement after timely efgartigimod initiation, further supporting its effectiveness in the induction phase.

Patient 12 did not achieve clinical improvement after three consecutive weeks of treatment, with no significant change in scale scores. This patient had chronic onset, was initially misdiagnosed, and only received immunotherapy eight months after symptom onset. Electromyography indicated demyelination with secondary axonal damage. The poor response is likely attributable to delayed diagnosis and treatment, leading to severe secondary axonal injury. In contrast, Patient 3 had a 14-year disease history but presented acutely initially, diagnosed with Guillain-Barré syndrome (GBS). Early treatment with IVIG and corticosteroids led to a good overall response despite relapses. Efgartigimod was chosen due to relapses after conventional therapy. At enrollment, symptoms were mild, and post-treatment score changes met the “improvement” criteria, indicating efficacy. Thus, patients receiving timely treatment early in the disease course, even with multiple relapses, may benefit more from efgartigimod. Furthermore, Patient 8 had severe symptoms at onset (INCAT score 10 at baseline) but, due to short disease duration and prompt diagnosis/treatment, showed marked improvement (INCAT score 2) one week after the fourth dose. Additionally, we observed that all A-CIDP patients in our cohort responded well and rapidly to efgartigimod: 71.4% met improvement criteria one week after the first dose, and 100% after four weeks. This may be related to their early diagnosis as GBS, prompt initiation of effective therapies like IVIG or PE, and relatively short disease duration at enrollment. CIDP is a chronic or relapsing-remitting inflammatory peripheral neuropathy requiring immunomodulatory therapy during active phases to prevent secondary axonal damage and permanent disability. Previous studies show that disability is associated with delayed initial treatment, which may also prolong overall treatment duration, increase complications, and impose greater economic burden (29–33). Therefore, early, effective, and sustained treatment is key to improving long-term CIDP prognosis.

This study employed both subcutaneous and intravenous administration, with six patients in each group. Analysis revealed no significant difference in score improvement between routes. Currently, there are no studies comparing the efficacy and safety of these two routes specifically for CIDP. However, the ADAPT NXT and ADAPT SC+ studies comparing IV and SC administration in MG are ongoing, and future clinical studies will further elucidate potential differences.

Efgartigimod is highly specific, reducing serum levels of all IgG subclasses. Other immunoglobulins are not FcRn-dependent, and efgartigimod targets FcRn, thus not affecting their levels. Compared to therapies like PE that remove all immunoglobulins, this translates to a potentially lower infection risk and a clearer safety advantage (15). In our study, except for one patient, IgG levels decreased post-treatment in all measured cases, while IgM and IgA showed no significant decrease. The exceptional patient had markedly lower baseline IgG. Although IgG levels changed little, clinical symptoms improved (INCAT score dropped to 0 after the fourth dose), suggesting efgartigimod is relatively safe and effective even in patients with low pre-treatment IgG. Albumin binds to FcRn at a site distinct from the IgG binding site. Efgartigimod is an IgG1 Fc fragment with minimal steric hindrance, not blocking albumin binding, thus not affecting albumin levels. We even observed increased albumin in some patients, similar to a previous case series report (22), though the reason remains unclear. Furthermore, previous studies on the FcRn inhibitor batoclimab showed significant LDL-C elevation, which was not observed in our study with efgartigimod.

The ADHERE study showed efgartigimod was well-tolerated and safe, with common treatment-emergent adverse events (TEAEs) including injection site reactions, headache, and infections, mostly mild or moderate. In our study, only one patient experienced a localized rash during one infusion, which resolved spontaneously. Our safety profile appears more favorable compared to RCTs, though the small sample size and short follow-up may influence this observation.

Compared to the ADHERE trial, real-world CIDP treatment is more complex, often involving concomitant immunosuppressants before or during efgartigimod therapy. In our study, most patients on combination therapy showed good efficacy and safety. Therefore, tailored combination therapy may benefit patients, though whether it is superior to efgartigimod monotherapy remains unclear.

This study has limitations. The sample size was small and from a single center, introducing potential selection bias. The wide age range and presence of comorbidities in some patients may affect efficacy assessment. The lack of a placebo control also impacts conclusion accuracy. All enrolled patients had typical CIDP, lacking observation of variant forms, which may influence drug efficacy evaluation. In practice, dose and interval adjustments based on individual circumstances may affect outcomes. Furthermore, the short follow-up period precludes assessment of long-term effectiveness and safety, Optimal dosage and interval for maintenance therapy remain undefined due to a lack of relevant studies or guidelines, necessitating further long-term follow-up to establish an appropriate regimen. Future studies with larger samples, longer follow-up, and controlled designs are needed to confirm our findings.

Currently, the use of efgartigimod for CIDP is still exploratory, requiring more clinical evidence. This study explored and validated the short-term efficacy and safety of efgartigimod for CIDP patients in a real-world setting, compared intravenous and subcutaneous formulations, and specifically noted the response in A-CIDP patients. It provides valuable experience for the practical clinical application of efgartigimod in CIDP and offers a reference for treating other antibody-mediated autoimmune diseases.

## Data Availability

The original contributions presented in the study are included in the article/**Supplementary Material**. Further inquiries can be directed to the corresponding authors.
