# Supplementary material for: A prospective real-world study of efgartigimod in the treatment of chronic inflammatory demyelinating polyradiculoneuropathy
Source: Front Immunol. 2026 Mar 2;17:1779544. doi: 10.3389/fimmu.2026.1779544 (PMC12989521; doi:10.3389/fimmu.2026.1779544)
Supplement: Supplementary file 1 [file Table1.docx]

Supplementary Material

**Supplementary Table S1** Changes in MRC Scores from Baseline After Treatment

| Time after treatment |  | All patients(n=12) | Intravenous(n=6) | Subcutaneous(n=6) | *P* value |
| --- | --- | --- | --- | --- | --- |
| 1 week |  | 3.42(6.75) | 1.50(1.98) | 5.33(9.35) | 0.349 |
| 2 weeks |  | 6.42(10.73) | 6.50(11.59) | 6.33(10.91) | 0.980 |
| 3 weeks |  | 8.08(12.88) | 8.50(13.77) | 7.67(13.23) | 0.917 |
| 4 weeks |  | 9.08(13.71) | 9.83(14.70) | 8.33(14.00) | 0.860 |

Data are mean (SD). Error bars show standard error.

**Supplementary Table S2.** Changes in I-RODS Scores from Baseline After Treatment

| Time after treatment | All patients(n=12) | Intravenous(n=6) | Subcutaneous(n=6) | *P* value |
| --- | --- | --- | --- | --- |
| 1 week | 7.00(9.51) | 4.83(4.12) | 9.17(13.06) | 0.468 |
| 2 weeks | 18.08(15.13) | 20.50(18.52) | 15.67(12.11) | 0.604 |
| 3 weeks | 21.92(16.08) | 24.00(18.63) | 19.83(14.53) | 0.675 |
| 4 weeks | 26.17(16.64) | 28.50(19.19) | 23.83(15.09) | 0.650 |

Data are mean (SD). Error bars show standard error.

**Supplementary Table S3.** Changes in INCAT Scores from Baseline After Treatment

| Time after treatment | All patients(n=12) | Intravenous(n=6) | Subcutaneous(n=6) | *P* value |
| --- | --- | --- | --- | --- |
| 1 week | -0.67(1.23) | -0.33(0.52) | -1.00(1.67) | 0.387 |
| 2 weeks | -2.08(2.50) | -2.17(3.06) | -2.00(2.10) | 0.915 |
| 3 weeks | -2.50(2.54) | -2.50(2.88) | -2.50(2.43) | 1.000 |
| 4 weeks | -3.00(2.73) | -3.17(2.99) | -2.83(2.71) | 0.844 |

Data are mean (SD). Error bars show standard error.

**Supplementary Table S4.** Changes in Immunoglobulin Levels Before and After Treatment

| Patient No. | IgG(g/L) | | IgM(g/L) | | IgA(g/L) | |
| --- | --- | --- | --- | --- | --- | --- |
|  | Start | last | Start | last | Start | last |
| P1 | 11.50 | 4.47 | 2.26 | 2.19 | 1.21 | 1.04 |
| P2 | 4.31 | 5.03 | 0.33 | 0.43 | 0.74 | 0.90 |
| P3 | 14.60 | 7.11 | 0.96 | 0.93 | 3.64 | 3.95 |
| P4 | 6.44 | 5.95 | 2.35 | 2.65 | 3.27 | 3.48 |
| P5 | 10.90 | 4.21 | 0.94 | 0.79 | 2.10 | 1.96 |
| P6 | 12.30 | 7.00 | 0.68 | 0.53 | 3.24 | 3.08 |
| P7 | 17.10 | 4.21 | 0.97 | 0.77 | 1.84 | 1.49 |
| P9 | 11.80 | 4.46 | 1.41 | 1.94 | 1.39 | 1.61 |
| P10 | 13.50 | 3.65 | 1.03 | 0.83 | 2.85 | 2.77 |

The patient numbering in this table corresponds to that in Table 2. Serum levels of IgG, IgM, and IgA were assessed before and after treatment in nine patients. “Start” denotes the time point immediately before the initial administration of efgartigimod, while “Last” indicates the time point at the final follow-up.
